# Wild Galois representations: elliptic curves with wild cyclic reduction

**DOI:** 10.1007/s40993-026-00730-5

**Published:** 2026-05-04

**Authors:** Nirvana Coppola

**Affiliations:** Dipartimento di Matematica, Università di Padova, Via Trieste 63, 35121 Padova, Italy

## Abstract

In 1990, Kraus [12] classified all possible inertia images of the $\ell$-adic Galois representation attached to an elliptic curve over a non-archimedean local field. In [1, 2], the author computed explicitly the Galois representation of elliptic curves having non-abelian inertia image, a phenomenon which only occurs when the residue characteristic of the field of definition is 2 or 3 and the curve attains good reduction over some non-abelian ramified extension. In this work, the computation of the Galois representation in all the remaining “wild” cases, i.e. when the residue characteristic is $p=2$ or 3 and the curve attains good reduction over an extension whose ramification degree is divisible by *p* (without assuming the condition on the image of inertia being non-abelian), is completed. This is based on Chapter V of the author’s PhD thesis [4].

## Introduction

Let *K* be a non-archimedean local field of mixed characteristic, or in other words, up to isomorphism, let *K* be a finite extension of $\mathbb {Q}_p$, for some rational prime *p*. Let *E* be an elliptic curve defined over *K*. For any prime $\ell$, one can consider the $\ell$-adic Tate module $T_\ell (E)$ and the action of the absolute Galois group ${{\,\textrm{Gal}\,}}(\overline{K}/K)$ of *K* on it, which defines a representation denoted by $\rho _{E,\ell }$. It is well-known that $\rho _{E,\ell }$, more precisely its restriction to the absolute inertia subgroup $I_K$ of ${{\,\textrm{Gal}\,}}(\overline{K}/K)$, encodes geometric and arithmetic properties of *E*: indeed the image $I=\rho _{E,\ell }(I_K)$ of inertia is

- trivial if and only if *E* has good reduction over *K*, by the Criterion of Néron-Ogg-Shafarevich [16, Theorem 1];
- finite if and only if *E* has potentially good reduction over *K*, as a consequence of the Criterion [16, Theorem 2];
- infinite if and only if *E* has potentially multiplicative reduction.

From an arithmetic point of view, the restriction to inertia of $\rho _{E,\ell }$ contains enough information to determine the conductor exponent of *E*, a nonnegative integer number which is nonzero if and only if the curve does not have good reduction, and can be defined for more general abelian varieties. Its computation is of interest, to name one example, for the application of the modular method. Indeed if an elliptic curve is modular (e.g. if it is defined over $\mathbb {Q}$, by [17]), then its conductor is equal to the level of the newform to which it is associated.

While the Galois representation in the cases of good and potentially multiplicative reduction were well understood (respectively by point-counting over the reduction of *E* [14, IV, §1.3] and by using the theory of Tate curves [15, V §5]) prior to the author’s PhD work, less was known about the potentially good case. For a survey of all the known cases, see also [4, §2].

If *E* has potentially good reduction, Kraus [12] classifies all possible inertia images *I* as abstract groups, in terms of invariants associated to *E*. The possibilities for the image of inertia can be summed up in the following finite list, where the notation is consistent with [8]:

- one of the following cyclic groups: $C_2,C_3,C_4,C_6$ (no restrictions on *p*);
- $C_3 \rtimes C_4$ (the dicyclic group), only for $p=3$;
- $Q_8$ (the quaternions) or ${{\,\textrm{SL}\,}}_2(\mathbb {F}_3)$, only for $p=2$.

Moreover, by [4, Theorem 2.10] or [16, Corollary 2 to Theorem 2], if $K^{nr}$ is the maximal unramfied extension of *K*, then *E* acquires good reduction over the minimal field $K^{nr}(E[m])$ where all *m*-torsion of *E* is defined, for $m\ge 3$ coprime to *p*. One thus has an explicit recipe to determine this extension, and thus the inertia image. Indeed, we have $I \cong {{\,\textrm{Gal}\,}}(K^{nr}(E[m])/K^{nr})$, see e.g. [2, §2].

If *E* has tame reduction, i.e. if it attains good reduction over a tamely ramified extension, the work of Dokchitser and Dokchitser [6] is enough to recover the full Galois representation. A survey of this is presented in [4], in a language that is compatible to that used in the present paper.

We say that *E* has wild reduction if it has potentially good reduction, which it attains over a wildly ramified extension. It follows that *E* has wild reduction if and only if *p* divides the order of *I*. The three non-abelian cases $C_3 \rtimes C_4$, $Q_8$ and ${{\,\textrm{SL}\,}}_2(\mathbb {F}_3)$, in particular, only occur as wild reduction cases. The large inertia image poses constraints on the whole $\rho _{E,\ell }$, making it relatively simple to determine it. This has been done by the author in [1, 2]. In this paper, the remaining wild cases are tackled. These are all cyclic, and unexpectedly more subtle than the non-abelian ones. By the list above, we know that if *E* has wild cyclic reduction and $p=3$, then $I \cong C_3$ or $C_6$, and if $p=2$, then $I \cong C_2, C_4$ or $C_6$. We will reduce these five possibilities to the study of the following two:

- $$p=3$$ and $$I \cong C_3$$,
- $$p=2$$ and $$I \cong C_4$$,

and give an explicit expression for $\rho _{E,\ell }$. In particular, in all these cases, there exists an explicit finite extension of *K* over which not only does *E* attain good reduction, but it is also possible to choose models over *K* with good reduction and residual curves:

- $$y^2=x^3-x$$, when $$p=3$$, and
- $$y^2+y=x^3$$, when $$p=2$$.

Using this and representation theory of finite groups, the representations are explicitly described in Theorem 3.1, for $p=3$, and in Theorem 4.3, for $p=2$.

The results presented in this paper are all included in Chapter V of the author’s PhD thesis [4].

We expect in the foreseeable future to implement this result in MAGMA, to complete the already existing functions “GaloisRepresentations” [3] containing the computation of $\rho _{E,\ell }$ in the non-abelian inertia cases, as in [1, 2].

As a final note, we mention the related work of [7], which aims at computing the so-called “inertial types” for elliptic curves defined over $\mathbb {Q}_p$. Although their approach and language is different from the one presented here, the motivation is the same.

## Notation

Throughout the paper, we will use the following notation.

**Table:** 

*p*	a rational prime
*K*	a *p*-adic field
*v*	a normalised valuation on *K*
$\pi$	a uniformiser of *K*
*k*	the residue field of *K*
$\overline{K}$	a fixed algebraic closure of *K*
$K^{nr}$	the maximal unramified extension of *K* in $\overline{K}$
*E*	an elliptic curve defined over *K*
$\Delta$	the discriminant of a given model of *E*
*E*[*m*]	the geometric *m*-torsion of *E*, for any integer *m*
$\ell$	a rational prime different from *p*
$T_\ell (E)$	the $\ell$-adic Tate module of *E*
$\rho _{E,\ell }$	the $\ell$-adic Galois representation of ${{\,\textrm{Gal}\,}}(\overline{K}/K)$ on $T_\ell (E)$
$I_K$	the inertia subgroup of ${{\,\textrm{Gal}\,}}(\overline{K}/K)$
*I*	the image of inertia $\rho _{E,\ell }(I_K)$

Moreover, we fix an isomorphism ${{\,\textrm{Aut}\,}}(T_\ell (E)) \cong \text {GL}_2(\mathbb {Z}_\ell )$ and, if $\overline{\mathbb {Q}}_\ell$ is an algebraic closure of $\mathbb {Q}_\ell$, we fix embeddings $\mathbb {Q}_\ell \hookrightarrow \overline{\mathbb {Q}}_\ell \hookrightarrow \mathbb {C}$. We still denote by $\rho _{E,\ell }$ the following complex representation:

$$ \rho _{E,\ell } : {{\,\textrm{Gal}\,}}(\overline{K}/K) \rightarrow {{\,\textrm{Aut}\,}}(T_\ell (E)) \cong \text {GL}_2(\mathbb {Z}_\ell ) \hookrightarrow \text {GL}_2(\mathbb {Q}_\ell ) \hookrightarrow \text {GL}_2(\mathbb {C}). $$

Let *F*/*K* be a totally ramified extension of *K*. Then, the quotient ${{\,\textrm{Gal}\,}}(\overline{K}/F)/I_F$ is isomorphic to the absolute Galois group of the residue field of *F* (that is equal to the residue field *k* of *K*, since *F*/*K* is totally ramified), which is generated, as a procyclic group, by the canonical Frobenius element, that acts as $x \mapsto x^{|k|}$. We fix a choice of a lift of the canonical Frobenius to ${{\,\textrm{Gal}\,}}(\overline{K}/F)$. Since ${{\,\textrm{Gal}\,}}(\overline{K}/F) \subseteq {{\,\textrm{Gal}\,}}(\overline{K}/K)$, we denote by ${{\,\textrm{Frob}\,}}_K \in {{\,\textrm{Gal}\,}}(\overline{K}/K)$ the element that we just defined, and by construction it is a Frobenius element of ${{\,\textrm{Gal}\,}}(\overline{K}/K)$ that fixes *F* point-wise.

## The case $p=3$

We assume for this section that $p=3$. Then, by [2, Theorem 2.7] and [12, Théorème 1], we have that *E* has wild cyclic reduction exactly when $v(\Delta )$ is even and *E* has Néron type different from $I_0^*$. By [2, Lemma 2.6] and [12, Corollaire du Lemme 3], this happens if and only if $v(\Delta )$ is even and *E* has no non-trivial 2-torsion points defined over $K^{nr}$. Fix a Weierstrass equation for *E*, of the form $y^2=f(x)$, and let $\alpha _1,\alpha _2,\alpha _3$ be the roots of *f* in $\overline{K}$. Let $F=K(\alpha _1)$. Then the Galois closure of *F*/*K* is *K*(*E*[2]).

If $v(\Delta ) \equiv 0 \pmod 4$, we fix ${{\,\textrm{Frob}\,}}_K$ to be a Frobenius element of ${{\,\textrm{Gal}\,}}(\overline{K}/K)$ that fixes *F* point-wise. If $v(\Delta ) \equiv 2 \pmod 4$ and *E* has type different from $I_0^*$, we fix ${{\,\textrm{Frob}\,}}_K$ to be the Frobenius element of ${{\,\textrm{Gal}\,}}(\overline{K}/K)$ that fixes *F* point-wise and a square root $\sqrt{\pi }$ of the uniformiser of *K*.

We will prove the following result.

### Theorem 3.1

Let *K* be a 3-adic field and let *E*/*K* have wild cyclic reduction. Let $\chi$ be the unramified character of ${{\,\textrm{Gal}\,}}(\overline{K}/K)$ that sends ${{\,\textrm{Frob}\,}}_K$ to $\sqrt{-|k|}$ (which we identify with $i \sqrt{|k|} \in \mathbb {C}$).

- If $v(\Delta ) \equiv 0 \pmod 4$ and $[K(E[2]):K]=6$, then $\rho _{E,\ell } = \chi \otimes \psi$, where $\psi$ is the compositum of the projection ${{\,\textrm{Gal}\,}}(\overline{K}/K) \rightarrow {{\,\textrm{Gal}\,}}(K(E[2])/K) \cong S_3$ with the unique irreducible 2-dimensional representation of $S_3$.
- If $v(\Delta ) \equiv 0 \pmod 4$ and $[K(E[2]):K]=3$, then:
  (i) if there are $\alpha _i$ and $\alpha _j$ such that $\alpha _i-\alpha _j$ is a square in *K*(*E*[2]), let $\psi$ be the order 3 character mapping any $\sigma \in I_K$ satisfying $\sigma (\alpha _j)=\alpha _i$ to $\dfrac{-1-\sqrt{-3}}{2}$. Then
    $$\begin{aligned} \rho _{E,\ell }=\chi \otimes \psi \oplus \overline{\chi } \otimes \overline{\psi }, \end{aligned}$$
    where $\overline{\bullet }$ denotes complex conjugation;
  (ii) otherwise, let $\sigma \in I_K$ permute cyclically the roots of *f*, with $\sigma (\alpha _1)=\alpha _2$, and let $\psi$ be defined analogously as in case (b.i). Then
    $$\begin{aligned} \rho _{E,\ell }=\overline{\chi } \otimes \psi \oplus \chi \otimes \overline{\psi }. \end{aligned}$$
- If $v(\Delta ) \equiv 2 \pmod 4$ and *E* has Néron type different from $I_0^*$, let $\chi _\pi$ be the quadratic character of $K(\sqrt{\pi })/K$. Then $E_\pi$, the quadratic twist of *E* by $K(\sqrt{\pi })$, has wild cyclic reduction and satisfies $v(\Delta ) \equiv 0 \pmod 4$. We have
  $$\begin{aligned} \rho _{E,\ell }=\rho _{E_\pi ,\ell } \otimes \chi _\pi . \end{aligned}$$

In the rest of this section, we prove each case separately.

### Proof of case (a)

First of all, *E* acquires good reduction over *F*. Indeed, we know by [16, Corollary 2 to Theorem 2] that it does over *K*(*E*[4]) and thus $\rho _{E,\ell }(I_{K(E[4])})$ is trivial, with $[I_K: I_{K(E[4])}]=3$, by [2, Theorem 2,7]. Moreover, $F \subseteq K(E[4])$ and it is totally ramified of degree 3 over *K*, so $[I_K: I_F]=3$. We conclude by the Criterion of Néron-Ogg-Shafarevich.

Now, as $[K(E[2]):K]=6$, we have that *K*(*E*[2])/*F* is quadratic and unramified. The same proof as the one for [4, Theorem 2.12], case *(b.ii)*, shows that $\rho _{E,\ell }({{\,\textrm{Frob}\,}}_K)$ has eigenvalues $\pm \sqrt{-|k|}$. Let $\chi$ be as in the statement. Then $\psi = \rho _{E,\ell } \otimes \chi ^{-1}$ factors through ${{\,\textrm{Gal}\,}}(K(E[2])/K)$, which is isomorphic to $S_3$. Since $\psi (I_K)=C_3$ acts faithfully, by direct inspection of the 2-dimensional representations of $S_3$ we deduce that $\psi$ is irreducible, so (as a representation of $S_3$) it is the unique 2-dimensional irreducible (and faithful) representation of $S_3$. $\square$

For the proof of case *(b.i)*, we assume first that $n=[k:\mathbb {F}_3]$ is odd. At the end of the proof, we will highlight what changes if *n* is even.

### Proof of case (b.i)

Assume that $n=[k:\mathbb {F}_3]$ is odd. Up to relabeling, suppose that $\alpha _2-\alpha _1$ is a square in $F=K(E[2])$, and fix a square root $\sqrt{\alpha _2-\alpha _1} \in F$. The following change of variables

$$\begin{aligned} \left\{ \begin{array}{lll} x & =&  (\alpha _2-\alpha _1)x'+\alpha _1, \\ y & =&  \sqrt{(\alpha _2-\alpha _1)^3} y' \end{array} \right. \end{aligned}$$

is defined over *F* and gives a model for the base change of *E* to *F* that reduces to $\tilde{E}: y^2=x^3-x$ over *k* (for the proof, see [2, Lemma 3.4]). Therefore, by [2, Example 2.5], the eigenvalues of $\rho _{E,\ell }({{\,\textrm{Frob}\,}}_K)$ are $\pm \sqrt{-|k|}$. We fix $\sigma \in I_K$ that permutes cyclically $\alpha _1,\alpha _2,\alpha _3$ and satisfies $\sigma (\alpha _1)=\alpha _2$, as in the statement. Then $\rho _{E,\ell }(\sigma )$ has determinant 1 (by the properties of the Weil pairing) and order 3, so it has eigenvalues given by the two primitive third roots of unity.

The image of $\rho _{E,\ell }$ is abelian, since it is isomorphic to ${{\,\textrm{Gal}\,}}(F^{nr}/K)$, which is the direct product of ${{\,\textrm{Gal}\,}}(F/K)$ and ${{\,\textrm{Gal}\,}}(K^{nr}/K)$. Therefore, we have a splitting of the form $\rho _{E,\ell }=\rho _1 \oplus \rho _2$, where the $\rho _i$’s are 1-dimensional. From the above, we know that, up to relabeling, $\rho _1({{\,\textrm{Frob}\,}}_K)=\chi ({{\,\textrm{Frob}\,}}_K)=\sqrt{-|k|}$, $\rho _2({{\,\textrm{Frob}\,}}_K)=\overline{\chi }({{\,\textrm{Frob}\,}}_K)=-\sqrt{-|k|}$ and $\rho _1(\sigma )$, $\rho _2(\sigma )$ are the two primitive third roots of unity, and it is only necessary to distinguish between the two.

The same argument as in the proof of [2, Theorem 1.1] (more precisely in Section 3.3) can be applied here, and it shows that $\rho _1(\sigma )=\dfrac{-1-\sqrt{-3}}{2}$, and $\rho _2(\sigma )=\dfrac{-1+\sqrt{-3}}{2}$. So, if we define $\psi : I_K \rightarrow \overline{\mathbb {Q}}_\ell ^\times$ such that $\psi (\sigma )=\dfrac{-1-\sqrt{-3}}{2}$, we have

$$\begin{aligned} \rho _{E,\ell } = \chi \otimes \psi \oplus \overline{\chi } \otimes \overline{\psi }, \end{aligned}$$

as claimed. $$\square $$

### Remark 3.2

If $n=[k:\mathbb {F}_3]$ is even, we still have the same good model for *E* over *F*, and we can compute the eigenvalues of Frobenius as in Example [2, Example 2.5], obtaining two identical real values, namely $(-3)^{n/2}$. In this case, we simply have $\rho _{E,\ell }=\chi \otimes (\psi \oplus \overline{\psi })$, for $\psi$ defined as in the statement. Since $\chi =\overline{\chi }$, we still recover $\rho _{E,\ell }=\chi \otimes \psi \oplus \overline{\chi } \otimes \overline{\psi }$.

### Proof of case (b.ii)

If all the $\alpha _i-\alpha _j$’s are not squares in $F = K(E[2])$, then $F(\sqrt{\alpha _2-\alpha _1})$ is quadratic and unramified over *F*. In fact, by assumption, $v(\Delta ) \equiv 0 \pmod 4$, and if $v_F$ is the normalised valuation on *F*, then $v(\Delta )=v_F(\Delta )/3=2v_F(\alpha _2-\alpha _1)$, since *E* has potentially good reduction. Therefore, $v_F(\alpha _2-\alpha _1)$ is even, so we can write $\alpha _2-\alpha _1=\pi _F^{2a}\epsilon$, where $\pi _F$ is a uniformiser of *F*, $a \in \mathbb {Z}$ and $\epsilon \in \mathcal {O}_F^\times$ is not a square. Moreover, we can take $\pi _F$ and $\epsilon$ so that $\epsilon \in \mathcal {O}_K^\times$. Let $E_\epsilon$ be the twist of *E* be $K(\sqrt{\epsilon })$. Then $E_\epsilon$ is as in case *(b.i)* of the theorem. In fact, since $K(\sqrt{\epsilon })/K$ is unramified, $E_\epsilon$ also has wild cyclic reduction over *K* with image of inertia $C_3$, and an equation for $E_\epsilon$ is precisely

$$\begin{aligned} y^2=(x-\epsilon \alpha _1)(x -\epsilon \alpha _2) (x-\epsilon \alpha _3), \end{aligned}$$

so $\epsilon \alpha _2-\epsilon \alpha _1 =\epsilon ^2 \pi _F^{2a}$ is a square in *F*.

By case *(b.i)*, we have $\rho _{E_\epsilon ,\ell } =\chi \otimes \psi \oplus \overline{\chi } \otimes \overline{\psi }$, where $\chi$ and $\psi$ are as in the statement. Let $\eta : {{\,\textrm{Gal}\,}}(\overline{K}/K) \rightarrow \{\pm 1\}$ be the unramified quadratic character of ${{\,\textrm{Gal}\,}}(\overline{K}/K)$. Then $\rho _{E_\epsilon ,\ell } = \rho _{E,\ell } \otimes \eta$, and an immediate computation shows that

$$\begin{aligned} \rho _{E,\ell }= \overline{\chi } \otimes \psi \oplus \chi \otimes \overline{\psi }, \end{aligned}$$

as claimed. $$\square $$

### Proof of case (c)

In this case, by [2, Theorem 2.7], we have $I=\rho _{E,\ell }(I_K) \cong C_6$. Let $E_\pi$ be the twist of *E* by $K(\sqrt{\pi })$. Then, the discriminant of $E_\pi$ is equal to $\pi ^6 \Delta$, and $v(\pi ^6 \Delta )=6+v(\Delta ) \equiv 0 \pmod 4$. Therefore, if $\chi _\pi$ is the quadratic character of ${{\,\textrm{Gal}\,}}(K(\sqrt{\pi })/K)$, we have $\rho _{E,\ell }=\rho _{E_\pi ,\ell } \otimes \chi _\pi$, where $\rho _{E_\pi ,\ell }$ is given by one of cases *(a)*, *(b.i)* or *(b.ii)*. $\square$

## The case $p=2$

We assume for this section that $p=2$. Recall that *E*/*K* has wild cyclic reduction exactly when the image of inertia $I=\rho _{E,\ell }(I_K)$ is one of $C_2$, $C_4$ or $C_6$, and [4, Theorem 2.9] and [12, Théorème 2, 3] classify these three cases.

We first consider the problem of the restriction to inertia of $\rho _{E,\ell }$ for $I \cong C_2$ or $C_6$. In order to do so, we start by viewing *E* as an elliptic curve over $K^{nr}$, since the reduction type and the action of inertia do not change. In particular, we have $\rho _{E,\ell }: {{\,\textrm{Gal}\,}}(\overline{K}/K^{nr}) \rightarrow {{\,\textrm{Aut}\,}}(T_\ell (E))$.

### Lemma 4.1

Let $E/K^{nr}$ be an elliptic curve with wild cyclic reduction and $I \not \cong C_4$. Then:

- if $I \cong C_2$, then *E* is a quadratic ramified twist of an elliptic curve with good reduction;
- if $I \cong C_6$, then *E* is a quadratic ramified twist of an elliptic curve with tame potentially good reduction.

The proof is essentially [7, Proposition 4.3].

### Proof

Assume first that $I \cong C_2$.

Let $L=K^{nr}(E[3])$. Then, by [2, Theorem 2.8] (or [16, §2 Corollary 3]), we have $I\cong {{\,\textrm{Gal}\,}}(L/K^{nr})$, so $L/K^{nr}$ is quadratic. Then the quadratic twist $E'$ of *E* by *L* has good reduction.

Assume now that $I \cong C_6$ and let *L* be as above. Then $L/K^{nr}$ is cyclic of order 6 and there is a unique quadratic subextension $M/K^{nr}$. The quadratic twist of *E* by *M* has tame potentially good reduction (achieved over *L*), with inertia image that is cyclic of order 3. $\square$

We now show that, in fact, there exists a quadratic twist of the base field *K*, over which *E* acquires good or tame reduction.

### Lemma 4.2

Let *E*/*K* be an elliptic curve with wild cyclic reduction and $I \not \cong C_4$. Then:

- if $I \cong C_2$ then *E* is a quadratic ramified twist of an elliptic curve with good reduction;
- if $I \cong C_6$ then *E* is a quadratic ramified twist of an elliptic curve with tame potentially good reduction.

### Proof

Assume that $I \cong C_2$ and let $L=K^{nr}(E[3])$ as in the proof of Lemma 4.1. Then ${{\,\textrm{Gal}\,}}(L/K)$ has inertia subgroup isomorphic to $C_2$, and the quotient is the procyclic group $\hat{\mathbb {Z}}$. Therefore, ${{\,\textrm{Gal}\,}}(L/K)$ is a semidirect product $C_2 \rtimes \hat{\mathbb {Z}}$, with $C_2$ normal in ${{\,\textrm{Gal}\,}}(L/K)$; but then the action of $\hat{\mathbb {Z}}$ can only be trivial, so in fact ${{\,\textrm{Gal}\,}}(L/K)= C_2 \times \hat{\mathbb {Z}}$. In particular, we can consider the intermediate extension *F*/*K* which is fixed by $\hat{\mathbb {Z}}$: this is Galois, quadratic and totally ramified, with *L*/*F* unramified, therefore *E* acquires good reduction over *F*.

If $I \cong C_6$, let *L* and *M* be as in the proof of Lemma 4.1, then ${{\,\textrm{Gal}\,}}(M/K)$ is isomorphic to the direct product $C_2 \times \hat{\mathbb {Z}}$ as in the previous case, and again by taking *F* to be the fixed field of $\hat{\mathbb {Z}}$ we conclude. $\square$

Notice that Lemma 4.2 shows the existence of a quadratic twist of *K* over which the curve acquires good or tame reduction, but it does not give an algorithmic result to compute it. Indeed, if $p=2$, there are several quadratic ramified extensions of *K* and we need to consider one such that its maximal unramified extension is equal to the field *L* in the proof of Lemma 4.2 above. Determining explicitly this extension is a non-trivial problem, which we do not tackle here. However, some partial explicit results are available if we restrict to $K=\mathbb {Q}_2$, namely [11, §4.1, Lemma 2].

Assuming we have computed a quadratic twist $E'/K$ of *E* with good or tame reduction, and if $\eta$ is the corresponding quadratic character, then $\rho _{E,\ell } = \rho _{E',\ell } \otimes \eta$, and $\rho _{E',\ell }$ is determined by [2, Lemma 2.4] (or [14, IV, §1.3]) if $I\cong C_2$ and [4, Theorem 2.12] case *(b)* if $I \cong C_6$.

For the rest of the section, we focus on the remaining wild cyclic case, that is $I \cong C_4$. We fix an arithmetic Frobenius element ${{\,\textrm{Frob}\,}}_K$ of ${{\,\textrm{Gal}\,}}(\overline{K}/K)$. We will specify which Frobenius we choose when the choice is relevant. Let $n=[k:\mathbb {F}_2]$ be the absolute inertia degree of *K*. We define the following unramified character of ${{\,\textrm{Gal}\,}}(\overline{K}/K)$:

$$\begin{aligned} \begin{array}{ll} \chi :&  {{\,\textrm{Gal}\,}}(\overline{K}/K) \rightarrow \overline{\mathbb {Q}}_\ell \hookrightarrow \mathbb {C}\\ &  {{\,\textrm{Frob}\,}}_K \mapsto (\sqrt{-2})^n \mapsto (i\sqrt{2})^n. \end{array} \end{aligned}$$

Let $G = {{\,\textrm{Gal}\,}}(K(E[3])/K)$. Then *G* is naturally embedded into $\text {GL}_2(\mathbb {F}_3)$, with the embedding given by fixing a basis for *E*[3] as an $\mathbb {F}_3$-vector space. We will show that $\rho _{E,\ell } \otimes \chi ^{-1}$ factors through *G*, and more precisely we will prove the following result.

### Theorem 4.3

Let $G={{\,\textrm{Gal}\,}}(K(E[3])/K)$ and suppose $I \cong C_4$. Then one of the following holds.

- $$G \cong C_4$$ and $$\rho _{E,\ell } = \chi \otimes (\psi \oplus \overline{\psi })$$, where
  $$\begin{aligned} \begin{array}{ll} \psi :&  G \rightarrow \overline{\mathbb {Q}}_\ell \hookrightarrow \mathbb {C}\\ &  \sigma \mapsto i \end{array} \end{aligned}$$
  for any fixed choice of a generator $\sigma$ of *G*;
- $G \cong Q_8$ or $D_4$ and $\rho _{E,\ell } = \chi \otimes \psi$, where $\psi$ is the only irreducible faithful 2-dimensional representation of *G*;
- $G\cong C_8$ and $\rho _{E,\ell } = \chi \otimes (\psi \oplus \overline{\psi })$, where $\psi$ is the faithful character of $C_8$ that maps a generator *g* of *G* that, seen as an element of $\text {GL}_2(\mathbb {F}_3)$, is $$\left( \begin{array}{cc} 2 & 2\\ 1 & 2 \end{array}\right) $$, to the 8-th root of unity $\dfrac{-\sqrt{2}+\sqrt{-2}}{2}$.

### Remark 4.4

Determining which of cases *(a)*, *(b)* or *(c)* occurs can be done, for instance, via [5, §3, Proposition 2 and Lemma 3].

### Proof

By definition, $I = \rho _{E,\ell }(I_K)$ is the image of the absolute inertia subgroup via $\rho _{E,\ell }$. Then we know, by [4, Theorem 2.10] or [16, Corollary 2 to Theorem 2], that $I \cong {{\,\textrm{Gal}\,}}(K^{nr}(E[3])/K^{nr})$, so *I* is isomorphic to the inertia subgroup of *G*. Therefore, *I* is a normal subgroup of *G* with cyclic quotient. By the classification in [5, §3, Proposition 2], it follows that there are only four possibilities for *G* to have $I \cong C_4$, namely *G* is one of $C_4,Q_8,D_4$ or $C_8$. In particular, in each of these cases *K* contains a third root of the discriminant of *E*, and we have that *n* is even if $G \cong C_4,Q_8$, while *n* is odd if $G \cong D_4$ or $C_8$.

Suppose first that $G \cong I \cong C_4$. Then *K*(*E*[3]) is a quartic extension of *K*, generated by the coordinates of one point of *E* of order 3, and following the proof of [1, Lemma 2.1] we obtain that there exists a model for the base change of *E* to *K*(*E*[3]) that reduces to $y^2+y=x^3$ over the residue field *k*. In particular, if we fix ${{\,\textrm{Frob}\,}}_K$ to be the arithmetic Frobenius that fixes *K*(*E*[3]) point-wise, we have that $\rho _{E,\ell }({{\,\textrm{Frob}\,}}_K)$ has eigenvalues $(\pm \sqrt{-2})^n$, and since *n* is even this means that $\rho _{E,\ell }({{\,\textrm{Frob}\,}}_K)$ is the scalar matrix $(-2)^{n/2} \text {Id}_2$. Therefore, $\rho _{E,\ell } \otimes \chi ^{-1}$ factors through $G\cong I$, and as a representation of *I* it is faithful with trivial determinant. By direct inspection on the character table of the group $C_4$ (see [9]), we deduce that $\rho _{E,\ell } \otimes \chi ^{-1}$ is the direct sum of the two one-dimensional faithful representations of $C_4$, hence it is $\psi \oplus \overline{\psi }$ where $\psi$ is as in the statement.

Now suppose that $G \cong Q_8$ or $D_4$. Then *G* is non-abelian, so by [5, §2, Lemma 1] we have that $\rho _{E,\ell } \otimes \chi ^{-1}$ factors through *G*, and as a representation of *G* it is irreducible and faithful. Since both $Q_8$ and $D_4$ have only one 2-dimensional irreducible representation (which is also faithful), case *(b)* of the theorem holds.

Finally, we assume that $G \cong C_8$. In this case, since *G* is cyclic, there exists a unique subextension of *K*(*E*[3]) of degree 2 over *K*, namely the unramified extension $K_2$ generated by a primitive third root of unity in $\overline{K}$, and $I \cong {{\,\textrm{Gal}\,}}(K(E[3])/K_2)$, with the restriction $\rho _{E,\ell } \big |_{I_K}$ factoring through *I*. By [13, Figure 4.3] we know that the 3-division polynomial of *E* over $K_2$ factors as the product of two quadratic factors. Let $x_P$ and $x_Q$ be the two different roots of one of these quadratic factors, and let $P, Q \in E[3] \setminus \{O\}$ have abscissa equal to $x_P$ and $x_Q$ respectively. Then $\{P,Q,-P,-Q\}$ is one *I*-orbit. This allows us to fix the following generator $\sigma$ of *I*: it is the element that acts on *E*[3] as

$$\begin{aligned} \left\{ \begin{array}{lll} P & \mapsto & Q, \\ Q & \mapsto & -P; \end{array} \right. \end{aligned}$$

so if we fix $\{P,Q\}$ as a basis for *E*[3] over $\mathbb {F}_3$, we identify $\sigma$ with the matrix

$$\begin{aligned} \left( \begin{array}{cc} 0 & 2\\ 1 & 0 \end{array}\right) . \end{aligned}$$

We now want to fix a generator *g* of *G*, and to do so we fix one of the two elements of order 8 in *G* with square equal to $\sigma$, namely $$g=\left( \begin{array}{cc} 2 & 2\\ 1 & 2 \end{array}\right) $$ as in the statement. Let us consider the representation $\rho _{E,\ell } \otimes \chi ^{-1}$. Again by the same proof as in [1, Lemma 2.1], we have that a model for the base change of *E* to *K*(*E*[3]) reduces to $y^2+y=x^3$, thus the Frobenius element of *K*(*E*[3]), which has inertia degree 2 over *K*, acts as the scalar matrix $(-2)^{n}\text {Id}_2$. We therefore have that the arithmetic Frobenius of *K* has distinct eigenvalues $\pm (\sqrt{-2})^n$, so $\rho _{E,\ell } \otimes \chi ^{-1}$ factors through *G*. We fix ${{\,\textrm{Frob}\,}}_K$ to be the Frobenius element of *K* that is mapped to *g* under the quotient map: ${{\,\textrm{Gal}\,}}(\overline{K}/K) \rightarrow G$. Moreover, the restriction to inertia of $\rho _{E,\ell } \otimes \chi ^{-1}$ factors through $I \cong C_4$, and as a representation of $C_4$ it is faithful with trivial determinant. Therefore $\rho _{E,\ell }\otimes \chi ^{-1} = \psi _a \oplus \psi _b$, where $\psi _a$ and $\psi _b$ are two of the four one-dimensional faithful representations of $C_8$, which are listed in [10]. Namely, the possible representations are denoted in op. cit. by $\rho _3, \rho _5, \rho _6, \rho _8$, and we identify *g* with the conjugacy class denoted by 8A, and the eighth root of unity $\zeta _8$ with the complex number $e^{2 \pi i /8}= \dfrac{\sqrt{2}+\sqrt{-2}}{2}$. Using that the restriction to inertia has trivial determinant, we deduce that the only possibilities for the set $\{\psi _a,\psi _b\}$ are:

$$\begin{aligned} \{\rho _3,\rho _5\}, \ \{\rho _3,\rho _8\}, \ \{\rho _5,\rho _6\}, \ \{\rho _6,\rho _8\}, \end{aligned}$$

and in particular $\psi _b = \overline{\psi _a}$. Let $\psi = \psi _a$. Now we have

$$\begin{aligned} \rho _{E,\ell } ({{\,\textrm{Frob}\,}}_K) = \chi ({{\,\textrm{Frob}\,}}_K) (\psi + \overline{\psi })(g). \end{aligned}$$

Let us fix $\ell = 3$. Then, the reduction modulo 3 of $\rho _{E,\ell }$ is equal to the modulo 3 Galois representation, i.e. the one given by the action of ${{\,\textrm{Gal}\,}}(\overline{K}/K)$ on *E*[3], so we have that $\rho _{E,\ell }({{\,\textrm{Frob}\,}}_K)$ reduces to $$g=\left( \begin{array}{cc} 2 & 2\\ 1 & 2 \end{array}\right) \pmod 3$$, which has trace 1 and determinant 2. A direct computation shows that the only pair in the list above for which this occurs is $\{\rho _6,\rho _8\}$, so the representation $\rho _{E,\ell }$ is given by

$$\begin{aligned} \rho _{E,\ell } = \chi \otimes (\rho _6 \oplus \rho _8), \end{aligned}$$

and this concludes the proof, since $\rho _6(g)=\zeta _8^3=\dfrac{-\sqrt{2}+\sqrt{-2}}{2}$. $\square$
